# Psychopathic traits and altered resting-state functional connectivity in incarcerated adolescent girls

**DOI:** 10.3389/fnimg.2023.1216494

**Published:** 2023-08-04

**Authors:** Corey H. Allen, J. Michael Maurer, Aparna R. Gullapalli, Bethany G. Edwards, Eyal Aharoni, Carla L. Harenski, Nathaniel E. Anderson, Keith A. Harenski, Vince D. Calhoun, Kent A. Kiehl

**Affiliations:** ^1^The Mind Research Network, Albuquerque, NM, United States; ^2^Department of Psychology, Georgia State University, Atlanta, GA, United States; ^3^Department of Electrical and Computer Engineering, Georgia Institute of Technology, Atlanta, GA, United States; ^4^Tri-Institutional Center for Translational Research in Neuroimaging and Data Science (TReNDS), Georgia State University, Georgia Institute of Technology, Emory University, Atlanta, GA, United States; ^5^Department of Computer Science, Georgia State University, Atlanta, GA, United States; ^6^Department of Psychology, University of New Mexico, Albuquerque, NM, United States

**Keywords:** psychopathic traits, functional connectivity, intra-network connectivity, spectra, ALFF, antisocial

## Abstract

Previous work in incarcerated boys and adult men and women suggest that individuals scoring high on psychopathic traits show altered resting-state limbic/paralimbic, and default mode functional network properties. However, it is unclear whether similar results extend to high-risk adolescent girls with elevated psychopathic traits. This study examined whether psychopathic traits [assessed via the Hare Psychopathy Checklist: Youth Version (PCL:YV)] were associated with altered inter-network connectivity, intra-network connectivity (i.e., functional coherence within a network), and amplitude of low-frequency fluctuations (ALFFs) across resting-state networks among high-risk incarcerated adolescent girls (*n* = 40). Resting-state networks were identified by applying group independent component analysis (ICA) to resting-state fMRI scans, and *a priori* regions of interest included limbic, paralimbic, and default mode network components. We tested the association of psychopathic traits (PCL:YV Factor 1 measuring affective/interpersonal traits and PCL:YV Factor 2 assessing antisocial/lifestyle traits) to these three resting-state measures. PCL:YV Factor 1 scores were associated with increased low-frequency and decreased high-frequency fluctuations in components corresponding to the default mode network, as well as increased intra-network FNC in components corresponding to cognitive control networks. PCL:YV Factor 2 scores were associated with increased low-frequency fluctuations in sensorimotor networks and decreased high-frequency fluctuations in default mode, sensorimotor, and visual networks. Consistent with previous analyses in incarcerated adult women, our results suggest that psychopathic traits among incarcerated adolescent girls are associated with altered intra-network ALFFs—primarily that of increased low-frequency and decreased high-frequency fluctuations—and connectivity across multiple networks including paralimbic regions. These results suggest stable neurobiological correlates of psychopathic traits among women across development.

## 1. Introduction

The construct of psychopathy is characterized as an array of traits, including callousness, impulsivity, poor decision-making, and a lack of empathy. These traits, alone, and in combination, have been found to be associated with poor interpersonal relationship success and treatment outcomes, and increased rates of substance use and rearrest (Taylor and Lang, 2006; Reidy et al., 2013; Mooney et al., 2019; Sohn et al., 2020; Allen et al., 2022a; Edwards et al., 2023). The societal cost of psychopathy to taxpayers is estimated to be nearly $460 billion per year, with $56.7 billion being accounted for by juveniles (Anderson, 1999; Caldwell et al., 2006; Kiehl and Hoffman, 2011; Cope et al., 2014; Reidy and Holland, 2018). Successful interventions for altering antisocial trajectories depend on gaining a better understanding of the underpinnings of such traits (Caldwell et al., 2006, 2007; Caldwell, 2011).

Research has identified a variety of causes and contributing conditions for psychopathic traits. Some of these include parenting style, childhood trauma, environmental exposures, and genetic make-up (Fergusson et al., 2008; Krischer and Sevecke, 2008; Wright et al., 2008; Marcus et al., 2010; Waller et al., 2012; Beckley et al., 2018; Sampson and Winter, 2018; Reuben et al., 2019). Brain imaging has shown that these traits are associated with altered functioning, primarily in limbic and paralimbic regions (e.g., insulae, temporal poles, posterior and anterior cingulate cortex, ventral striatum, and amygdalae) but also across the default mode network more generally [DM: e.g., precuneus and medial prefrontal cortex (mPFC): Chen et al., 2015; Cohn et al., 2015; Thijssen and Kiehl, 2017; Dugré and Potvin, 2021; Thijssen et al., 2021; Umbach and Tottenham, 2021; Werhahn et al., 2021; Winters et al., 2021]. While the literature investigating the relationship between functional connectivity and antisocial traits in adolescents is growing, there is an absence of research focusing specifically on high-risk adolescent girls with established poor behavioral outcomes (i.e., arrests and convictions). Prior studies have focused mainly on boys, leaving potential sex-specific developmental differences corresponding to psychopathic traits unexplored. With rates of incarceration of adolescent girls declining more slowly than those of boys (U.S. Department of Justice, Office of Justice Programs, 2021), and rates of incarceration of girls generally increasing on a global scale (Reynolds, 2008; Harmon and Boppre, 2018), the impetus to fill this gap is evident.

Because of the relatively sparse literature concerning resting-state alterations in adolescent girls relating to psychopathic traits, it is unclear whether alterations are stable from adolescence to adulthood, or rather, present differently in younger samples. Our research group recently explored resting-state alterations in adult women scoring high on psychopathic traits. We found that interpersonal/affective psychopathic traits (e.g., deficient empathy, a lack of remorse, and manipulativeness) were associated with increased amplitude of low-frequency fluctuations (ALFFs) in executive control and attentional networks, decreased high-frequency ALFFs in executive control and visual networks, and decreased intra-network connectivity in the default mode network. Lifestyle/antisocial psychopathic traits were associated with decreased high-frequency ALFFs in executive control and default mode networks, and both increased and decreased intra-network functional connectivity in visual networks (Allen et al., 2022b), diverging from similar analyses conducted in adult men scoring high on psychopathic traits which found no effects for the same measure (Espinoza et al., 2018). These results suggest potentially sex-specific neurobiological correlates of high-risk phenotypes, primarily occurring across regions and networks involved in socioemotional processing. Identifying stable or divergent neurobiological alterations in adolescent girls compared to those in adult women may inform our understanding of possible intervention targets designed to reduce psychopathic traits.

The relationship of psychopathic traits to other resting-state activational measures in incarcerated adolescent girls, including ALFFs, has been left unexplored. Due to their demonstrated association with functional connectivity more generally, and psychiatric disorders and behavioral characteristics, ALFFs may be a useful mode of investigation in relation to psychopathic traits (Guo et al., 2013; Yue et al., 2015; Wielaard et al., 2018; Eggart et al., 2019; Weightman et al., 2019; Zamani Esfahlani et al., 2020; Allen et al., 2022b; Gazula et al., 2022). By assessing the relationship of psychopathic traits to measures previously examined in the literature—such as altered inter-network and intra-network functional connectivity—as well as those unexamined in the literature (ALFFs), a more thorough picture of how these traits relate to altered functional brain connectivity on a local and global scale in relation to adolescent girls can be offered.

Here we examine resting-state metrics and their relationships to psychopathic traits [assessed via the Hare Psychopathy Checklist: Youth Version (PCL:YV)] (Forth et al., 2003) in a sample of incarcerated adolescent girls (*n* = 40). Resting networks were assessed using three different metrics [static functional network connectivity (sFNC: inter-network connectivity), ALFFs, and intra-network connectivity], to comprehensively evaluate local and global associations with psychopathic traits in incarcerated adolescent girls. We hypothesized that abnormalities in functional network connectivity related to psychopathic traits would occur primarily in limbic, paralimbic, and default mode network related regions of the brain (i.e., temporal poles, amygdalae, caudate/putamen, orbitofrontal cortex, dorsomedial prefrontal cortex, posterior cingulate cortex, and precunei). These regions span multiple cognitive domains, are involved in higher-order cognitive processes, such as emotion regulation, and are consistent with previously published studies in adolescent boys and adult men and women (Kiehl, 2006; Cope et al., 2014; Fairchild et al., 2014; Chen et al., 2015; Cohn et al., 2015; Philippi et al., 2015; Thijssen and Kiehl, 2017; Lindner et al., 2018; Dugré and Potvin, 2021; Thijssen et al., 2021; Umbach and Tottenham, 2021; Werhahn et al., 2021; Winters et al., 2021; Allen et al., 2022b). Parallel to analyses by our research group in incarcerated adult women and adolescent boys scoring high on psychopathic traits, this study serves an important role in assessing whether psychopathy-related neural alterations are consistent from adolescence to adulthood, or rather, present differently in younger samples of women.

## 2. Methods

### 2.1. Participants

Participants included adolescent girls from the National Institute of Mental Health (NIMH)-funded SouthWest Advanced Neuroimaging Cohort, Youth sample (SWANC-Y), at a maximum-security juvenile correctional facility in New Mexico, collected between June 2007 and March 2011. With an initial sample of *n* = 78, exclusions included participants that did not have a resting-state scan (*n* = 27), or PCL:YV administered (*n* = 7), and poor brain masks during scan (*n* = 4), leaving complete data sets from 40 incarcerated adolescent girls, ranging from 14 to 20 years of age.^1^ The average age of participants was 17.4 years (SD = 1.0: see Table 1). Using National Institutes of Health racial and ethnic classification, 45.0% of the sample self-identified as white, 7.5% as black/African American, 25.0% as American Indian or Alaskan Native, 22.5% as multiracial/other, and, ethnically, 60% as Hispanic or Latina. 97.5% participants were right-handed.

**Table 1:** Participant demographics and assessment scores.

	**Mean**	***SD***	**Min**.	**Max**.	**Overall sample (%)**
Age (years)	17.4	1.0	14.8	20.0	
IQ	98.1	11.3	66	126	
PCL:YV total scores	23.0	6.1	11.0	36.0	
Factor 1 scores	7.2	3.3	1.0	13.0	
Factor 2 scores	14.4	3.0	6.0	19.0	
SUD	2.6	1.6	0	7	92.3
Mood					38.5
Anxiety					12.8
PTSD					28.2
ADHD					15.4

Endorsement of psychiatric disorder reflects a participant meeting past or present criteria for any mood or anxiety disorder, and PTSD/ADHD singularly (n = 39).

Participants provided written informed consent (if ≥18 years or age) or written informed assent and parent/guardian written informed consent (if <18 years of age), in protocols approved by the Institutional Review Board of the University of New Mexico and were paid at a rate commensurate with institution compensation for work assignments at the correctional facility.

### 2.2. Assessments and measures

#### 2.2.1. Psychopathic traits

Psychopathic traits were assessed using the Psychopathy Checklist: Youth Version (PCL:YV; Forth et al., 2003). The assessment includes a semi-structured interview covering individuals' school, family, work, and antisocial histories, as well as their interpersonal and emotional skills and a review of institutional records. Individuals are scored from zero to two on 20 different items that measure traits and behavioral characteristics of psychopathy, with total scores ranging from zero to 40 (see Kosson et al., 2002, 2013 for further assessment information). Interviews were conducted by trained researchers and videotaped for reliability assessment. Consistent with the literature (Thijssen and Kiehl, 2017), and in addition to PCL:YV Total scores, we examined a two-factor model of psychopathic traits (Harpur et al., 1989; Hare, 2003; Kennealy et al., 2007). The two-factor model of psychopathic traits was originally constructed via factor analysis (Harpur et al., 1989; Hare, 1991). Two correlated overarching factors that held explanatory value for the underlying individual items. Subsequent confirmatory factor analyses suggest each of these factors can be further explained by two underlying facets (Vitacco et al., 2005; Kosson et al., 2013). Specifically, Factor 1 is composed of interpersonal and affective facets (e.g., grandiosity and a lack of empathy), whereas Factor 2 is composed of antisocial and developmental facets (e.g., impulsivity and early behavioral problems). While the number of factors to extract for the PCL:YV is an open debate (Kosson et al., 2013), we chose to focus on the two-factor model for a more direct comparison to previous analyses in adult women and adolescent boy samples (see Thijssen and Kiehl, 2017; Allen et al., 2022b). An analysis of all PCL:YV items within this sample suggest high internal reliability (Cronbach's alpha = 0.82).

#### 2.2.2. IQ

Participants' IQs were estimated from the Wechsler Intelligence Scale for Children-Fourth Edition (WISC-IV; Wechsler, 2003; Sattler and Dumont, 2004) for those younger than 16 years of age and from the Vocabulary and Matrix Reasoning subtests of the Wechsler Adult Intelligence Scale (WAIS-III; Wechsler, 1997; Ryan and Ward, 1999) for those older than 16 years of age. The mean full-scale IQ estimate in this sample was 98.1 (SD = 11.3: see Table 1); IQ scores were unavailable for eight participants and were subsequently mean replaced for inclusion in imaging analyses.

#### 2.2.3. Diagnosis of psychiatric disorders

To assess whether or not participants met criteria for various forms of psychopathology, including mood disorders, anxiety disorders, post-traumatic stress disorder (PTSD), and attention-deficit/hyperactivity disorder (ADHD), we utilized the Kiddie Schedule for Affective Disorders and Schizophrenia (KSADS; scoring and criteria explained in Kaufman et al., 1997). Categorization of potential mood disorders included major depressive disorder (with and without psychotic features), melancholic depression, dysthymia, adjustment disorder with depressed mood, depressive disorder NOS, schizoaffective disorder (depressed and manic types), mania, hypomania, cyclothymia, or bipolar disorder NOS. Anxiety disorders included obsessive compulsive disorder, generalized anxiety disorder, acute stress disorder, panic disorder (with and without agoraphobia), separation anxiety, phobias (i.e., social phobia and/or specific phobias), agoraphobia, or an anxiety disorder not otherwise specified (NOS). Based on this criteria, and out of the 39 participants that were administered the KSADS, 15 participants met criteria for any mood disorder, five participants met criteria for any anxiety disorder, six met criteria for ADHD, and 11 participants met criteria for PTSD (see Table 1).

#### 2.2.4. Substance use

For descriptive purposes, and similarly to other published methods (Cope et al., 2014; Edwards et al., 2023), we assessed substance use history using the KSADS, summing the total number of substances (alcohol, cannabis, sedatives/hypnotics/anxiolytics, cocaine, opioids, hallucinogens, stimulants, and solvents/inhalants/other) for which an individual met the lifetime dependence diagnostic criteria was calculated [substance dependence (SUD); theoretical range: 0–8, M = 2.6, SD = 1.6]. A dimensional score for SUD was used to provide a more meaningful and representative measure of substance use for the sample, as ~92% of participants met criteria for at least one SUD (see Table 1).

### 2.3. Imaging parameters

Resting-state functional magnetic resonance images were collected at the correctional facility where participants were housed, using the Mind Research Network's mobile Siemens 1.5T Avanto with advanced SQ gradients (max slew rate 200T/m/s, 346T/m/s vector summation, rise time 200 μs) equipped with a 12-element head coil. The EPI gradient echo pulse sequence (TR = 2,000 ms, TE = 39 ms, flip angle = 75, FOV = 24 × 24 cm, 64 × 64 matrix, 3.75 × 3.75 mm in-plane resolution, 4 mm slice thickness, 1 mm gap, 27 slices) effectively covered the entire brain (150 mm) in 2.0 s. Head motion was minimized using padding and restraint. The participants were asked to lay still, look at the fixation cross, and keep eyes open during the 5-min rsfMRI scanning.

### 2.4. EPI preprocessing

Data were preprocessed using statistical parametric mapping (SPM12) (Friston et al., 1994) (http://www.fil.ion.ucl.ac.uk/spm) including image reorientation, realignment [motion estimation using INRialign (Freire and Mangin, 2001)], and spatial normalization to the Montreal Neurological Institute standard space at a resolution of a 3 × 3 × 3 mm^3^. A full width half maximum Gaussian kernel of 6 mm was then used for spatial smoothing. Framewise displacement (FD) was used to assess motion quality control. For FD, the translation and rotation parameters were computed as the mean of the sums of the absolute translation and rotation frame displacements. Following the removal of participants with scans resulting in inadequate brain masks (i.e., those missing large areas of brain), all participants demonstrated a mean FD < 0.3 mm, and therefore, none were removed due to excessive motion (Stout et al., 2021). Additionally, ArtRepair was used to remove noise spikes larger than 4% of the global signal (Mazaika et al., 2007), further addressing the management of subject motion.

### 2.5. Independent component analysis

**Figure 1:**
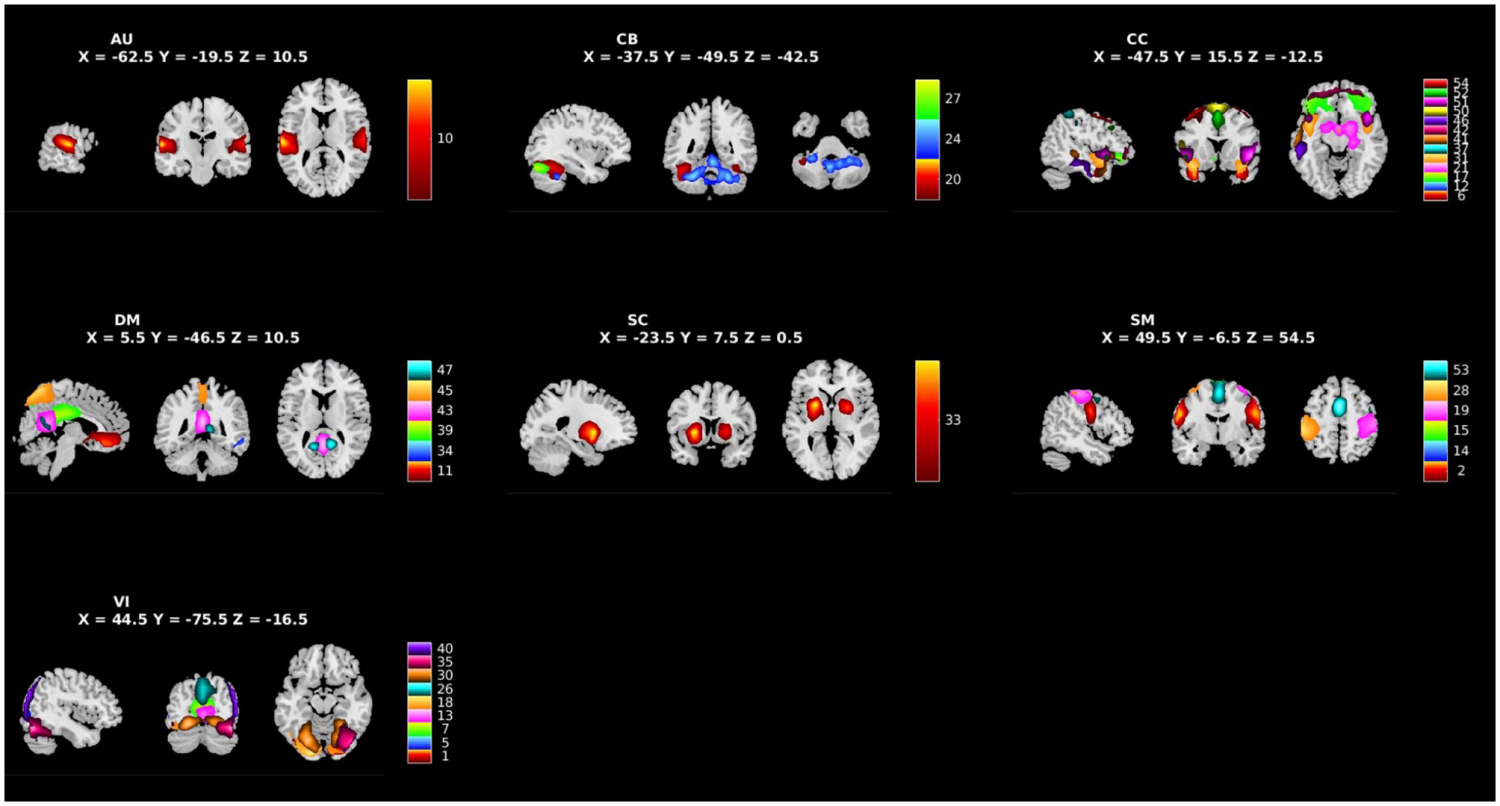
Spatial maps of the 39 independent components identified as RSNs categorized by domain [auditory (AU), cerebellar (CB), cognitive control (CC), default mode (DM), subcortical (SC), sensorimotor (SM), and visual (VI)] and component number.

**Figure 2:**
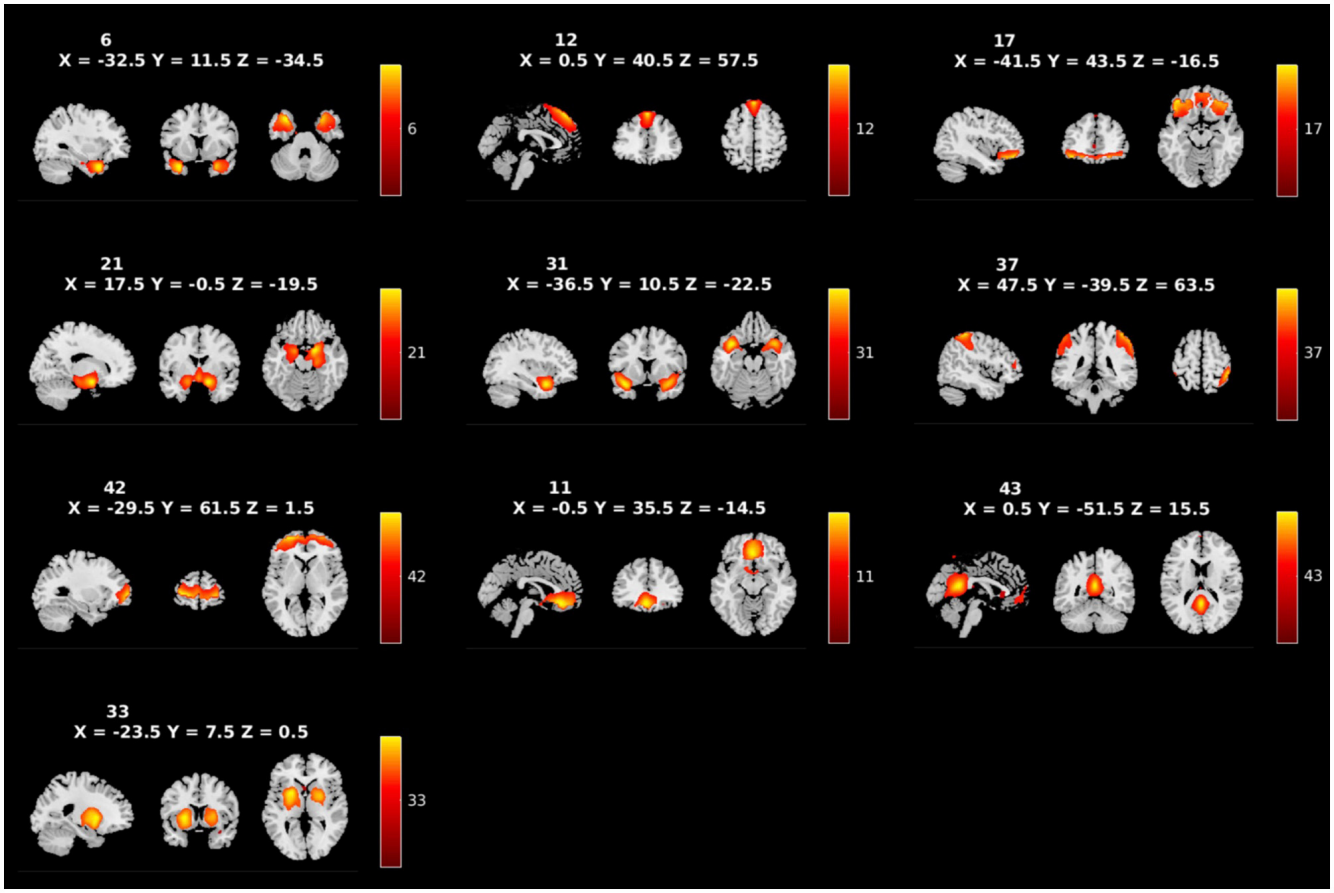
Spatial maps of the 10 RSNs identified as *a priori* networks of interest, labeled by component number. Broadly, the networks of interest include the temporal pole (ICs 6 and 31), the dmPFC (IC12), the pars orbitalis (IC17), the amygdalae (IC21), the precuneus (IC37), the aPFC (IC42), the OFC (IC11), the PCC (IC43), and the caudate/putamen (IC33).

We applied group ICA (gICA) on the preprocessed rsfMRI data using the Group ICA of fMRI Toolbox (GIFT: http://trendscenter.org/software/gift) (Calhoun et al., 2001). The rsfMRI data was compressed using two stages of principal component analysis (PCA) (Rachakonda et al., 2016). Consistent with previously published studies, in the first step of data reduction, we retained 100 principal components (PCs), and 75 independent components (ICs) for group data reduction, (Kiviniemi et al., 2009; Smith et al., 2009; Ystad et al., 2010; Abou Elseoud et al., 2011; Allen et al., 2011a; Erhardt et al., 2011). High-model order ICA (i.e., 75 components) yields more refined components that correspond more closely to known functional and anatomical segmentations in comparison to low-model order ICA (i.e., 25 or 50 components) (Allen et al., 2011a; Hu et al., 2020). Participant specific spatial maps and their corresponding time-courses were obtained using gICA. Out of the 75 ICs that were estimated, 39 components were identified as components of RSNs by evaluating whether peak network activation occurred in gray matter and whether the peak ALFFs occurred in the low-frequency power portion of the spectra of components (see Figure 1 for whole-brain component solution: Meda et al., 2008; Robinson et al., 2009; Allen et al., 2011b). The other 36 components were excluded, as they appeared to be related to motion artifacts, the ventricular system, or cerebrospinal fluid, spatial maps including white matter, or having irregular time-course spectra power or low stability (Allen et al., 2011b). The reliability and stability of these extracted networks were evaluated via ICASSO (Himberg and Hyvarinen, 2003), a process that iteratively re-runs component estimations with alternatively bootstrapped datasets. This analysis suggested high stability across the selected 39 components (mean stability index = 0.94), well above the threshold of 0.80 established in the literature (Ma et al., 2011). Within GIFT, the time-courses of the RSNs underwent despiking and bandpass filtering with (0.01–0.15) Hz cutoffs. From the 39 extracted components, 10 components of interest were selected for the primary analyses based on relevant literature (see Figure 2 for *a priori* components of interest: Kiehl, 2006; Cope et al., 2014; Fairchild et al., 2014; Chen et al., 2015; Cohn et al., 2015; Philippi et al., 2015; Thijssen and Kiehl, 2017; Lindner et al., 2018; Dugré and Potvin, 2021; Thijssen et al., 2021; Umbach and Tottenham, 2021; Werhahn et al., 2021; Winters et al., 2021; Allen et al., 2022b).

### 2.6. Resting-state measures

In order to assess various types of resting-state functional connectivity and activational measures, using the GIFT toolbox and its suggested default parameters (http://trendscenter.org/software/gift) (Calhoun et al., 2001), we calculated the sFNC between the selected RSNs as pairwise correlations between the RSNs' time-courses for each individual (inter-network connectivity), pairwise correlations between individual voxels within the RSN to the overall RSN's time-course (intra-network connectivity), and the Fourier transform of individual RSN time-courses (ALFFs: decompositions of the time-course into the frequencies of activation and their amplitudes).

### 2.7. Statistical analyses

We performed regression analysis to identify associations between sFNC values (inter-network connectivity), spatial maps (intra-network connectivity), and ALFFs with psychopathy measures: PCL:YV Factor 1, Factor 2, and Total scores, which were included as continuous variables. The analyses were corrected for “nuisance” covariates (age at scan and IQ). Univariate associations between psychopathic traits and *a priori* networks of interest were first examined. For these region of interest analyses, we report both uncorrected and false discovery rate (FDR) corrected results, at an alpha level of 0.05 (Genovese et al., 2002; Thijssen and Kiehl, 2017). Additionally, we performed exploratory whole-brain analyses (i.e., tested all extracted components rather than solely *a priori* networks of interest) using FDR multiple comparison correction.

## 3. Results

### 3.1. Psychopathic traits

The PCL:YV total scores for this sample ranged from 11.0 to 36.0 (*M* = 23.0, SD = 6.1), the PCL:YV Factor 1 scores ranged from 1.0 to 13.0 (*M* = 7.2, SD = 3.3), and the PCL:YV Factor 2 scores ranged from 6.0 to 19.0 (*M* = 14.4, SD = 3.0; see Table 1 for descriptive statistics and Supplementary Table S1 for correlations with other psychiatric data).

### 3.2. Group independent component analysis and *a priori* component selection

Figure 1 shows the spatial maps of the 39 RSNs across the whole brain. The 39 RSNs listed in Supplementary Table S2 were grouped into seven domains: auditory (AU), cerebellar (CB), cognitive control (CC), default mode network (DM), subcortical (SC), sensorimotor (SM), and visual (VI) based on their peak coordinate, functional properties, the automatic labeling tool in GIFT (see Supplementary Figure S1 for average sFNC across all components and domains, suggesting high intra-domain sFNC: Du et al., 2020; Salman et al., 2022), and confirmed by visual inspection. From these 39 components, 10 components were selected as *a priori* candidates for analysis (see Figure 2: Philippi et al., 2015; Thijssen and Kiehl, 2017; Espinoza et al., 2018; Lindner et al., 2018; Allen et al., 2022b).

### 3.3. Time-course power spectra

#### 3.3.1. PCL:YV Factor 1 scores

**Figure 3:**
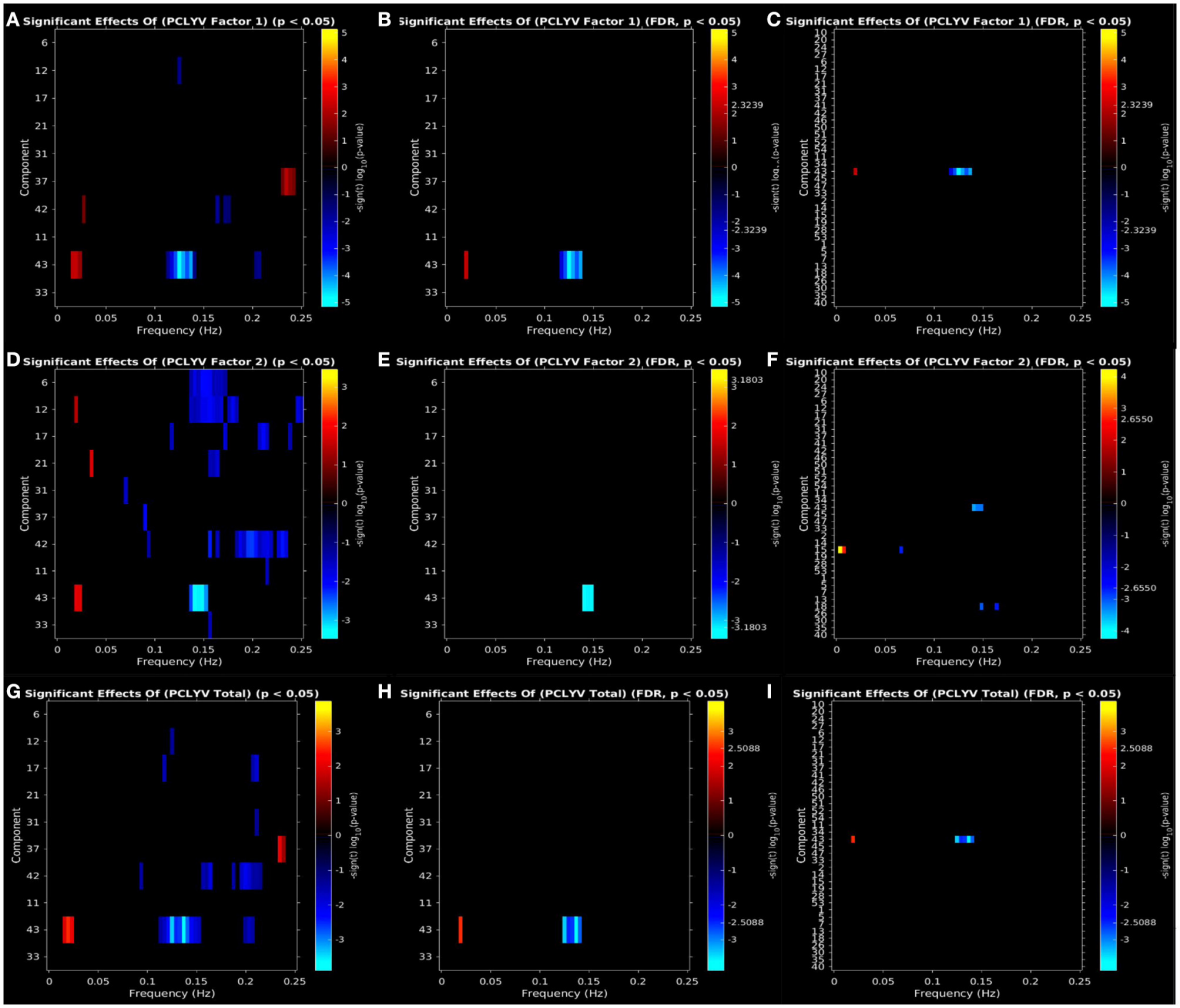
Univariate associations between psychopathic traits [**(A–C)**: PCL:YV Factor 1, **(D–F)**: PCL:YV Factor 2, **(G–I)**: PCL:YV Total] and power spectra (ALFFs) of the significant components. Panel depicts the significance and direction of effects as a function of frequency for the significant components, displayed as–sign(*t*) log_10_(*p*), at an **(A**, **D**, **G)** uncorrected threshold of *p* < 0.05 and **(B**, **E**, **H)** FDR corrected *p* < 0.05 for *a priori* networks of interest, and **(C, F, I)** FDR corrected *p* < 0.05 for the exploratory whole-brain analysis.

PCL:YV Factor 1 scores were associated with increased ALFF at low-frequency bands (0–0.05 Hz) in the aPFC (IC 42, CC) and PCC (IC43, DM), decreased ALFF at mid/high-frequency bands (0.10–0.20 Hz) in the dmPFC (IC12, CC), aPFC (IC 42, CC), and PCC (IC43, DM), and increased ALFF at high-frequency bands (0.20–0.25 Hz) in the precuneus (IC37, CC: see Figure 3A). Effects of increased ALFF at low-frequency bands (0–0.05 Hz) and decreased ALFF at mid-frequency bands (0.10–0.15 Hz) in the PCC (IC43, DM) remain following FDR correction and also emerge in the exploratory whole-brain analysis (see Figures 3B, C).

#### 3.3.2. PCL:YV Factor 2 scores

PCL:YV Factor 2 scores were associated with increased ALFF at low-frequency bands (0–0.05 Hz) in the dmPFC (IC12, CC), amygdalae (IC21, CC), and PCC (IC43, DM), and decreased ALFF at mid/high-frequency bands (0.10–0.25Hz) in the temporal pole (IC6 and IC31, CC), dmPFC (IC12, CC), pars orbitalis (IC17, CC), amygdalae (IC21, CC), precuneus (IC37, CC), aPFC (IC 42, CC), OFC (IC11, DM), PCC (IC43, DM), and caudate/putamen (IC33, SC: see Figure 3D). Effects of decreased ALFFs at mid-frequency bands in the PCC (IC43, DM) remain following FDR correction and also emerge in the exploratory whole-brain analysis (see Figures 3E, F). Additionally, the whole-brain analysis also suggests a relationship between PCL:YV Factor 2 scores and decreased ALFFs at mid-frequency bands (0.05–0.20 Hz) in the primary motor cortex (IC15, SM) and secondary visual cortex (IC18, VI), and increased ALFFs at low-frequency bands (0–0.01 Hz) in the primary motor cortex (IC15, SM: see Figure 3F).

#### 3.3.3. PCL:YV Total scores

PCL:YV Total scores were associated with increased ALFF at low-frequency bands (0–0.05 Hz) in the PCC (IC43, DM), decreased ALFF at mid/high-frequency bands (0.10–0.25 Hz) in the temporal pole (IC31, CC), dmPFC (IC12, CC), pars orbitalis (IC17, CC), aPFC (IC 42, CC), and PCC (IC43, DM), and increased ALFF at high-frequency bands (0.20–0.25 Hz) in the precuneus (IC37, CC: see Figure 3G). Effects of increased ALFFs in low-frequency and decreased ALFFs at mid-frequency bands in the PCC (IC43, DM) remain following FDR correction and also emerge in the exploratory whole-brain analysis (see Figures 3H, I).

### 3.4. Intra-network connectivity

#### 3.4.1. PCL:YV Factor 1 scores

**Figure 4:**
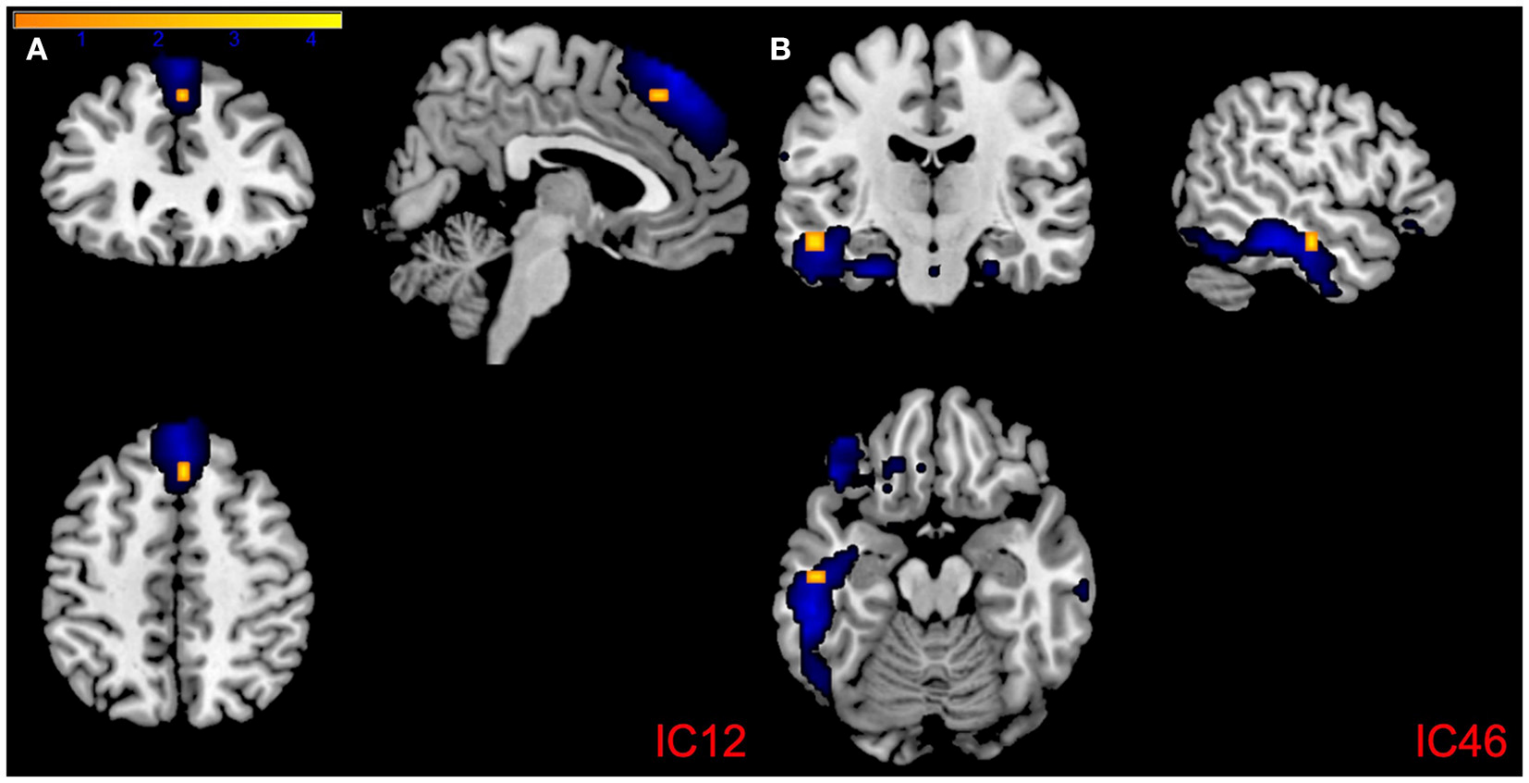
Regions of intra-network connectivity effects for visualization purposes. **(A)** Association between PCL:YV Factor 1 score and intra-network connectivity within component 12 and **(B)** component 46, FDR corrected *p* < 0.05. Blue mapping corresponds to component 12 and 46's spatial maps and orange reflects regions of increased intra-network connectivity, with the color bar indicating *T*-values ranging from 0 to 4.3.

PCL:YV Factor 1 scores were associated with altered intra-network connectivity in regions within all *a priori* networks of interest (see Table 2). Specifically, Factor 1 related increases of intra-network connectivity were found in the middle, and superior temporal gyrus, middle, medial, and inferior frontal gyrus, caudate, insula, parahippocampal gyrus, and lentiform nucleus. Factor 1 related decreases of intra-network connectivity were found in the superior temporal gyrus, parahippocampal gyrus, medial and inferior frontal gyrus, insula, caudate, anterior cingulate, and the precuneus (see Table 2). Effects of increased intra-network connectivity in the medial frontal gyrus relative to the dmPFC (IC12, CC) remain following FDR correction and also emerge in the exploratory whole-brain analysis (see Figure 4A, Table 2). Additionally, the whole-brain analysis also suggests a relationship between PCL:YV Factor 1 scores and increased intra-network connectivity in the middle temporal gyrus relative to the inferior temporal gyrus (see Figure 4B, Table 2).

**Table 2:** Effects of psychopathic traits on intra-network connectivity.

**Region**	**Hemisphere**	**Max *T*-value**	**MNI coordinates *x*, *y*, *z***
**PCL:YV Factor 1 intra-network connectivity effects: positive**			
Middle temporal gyrus	L	4.3^†^	−51, −16, −17
Medial frontal gyrus	L	2.8	−9, 56, −2
	R	3.9^†^^*^	3, 29, 49
Caudate	R	3.5	21, 20, 7
	R	2.4	18, 17, 7
Inferior frontal gyrus	L	3.5	−30, 26, −14
Superior temporal gyrus	L	2.8	−51, 11, −20
Insula	L	2.4	−42, −13, −8
	R	2.0	36, −7, −14
Parahippocampal gyrus	R	2.2	24, −19, −17
Middle frontal gyrus	R	2.1	39, 44, −8
Lentiform nucleus	R	2.1	27, −7, −8
**PCL:YV Factor 1 intra-network connectivity effects: negative**			
Superior temporal gyrus	L	3.5	−30, 11, −41
	R	2.2	51, −10, −2
	L	2.3	−51, −7, −11
Parahippocampal gyrus	L	3.1	−21, −7, −29
Medial frontal gyrus	L	2.5	−9, 38, 40
	R	2.9	9, 38, 43
Insula	L	2.5	−39, 11, −8
Caudate	R	2.8	15, 5, 7
Anterior cingulate	L	2.5	−6, 44, −5
Inferior frontal gyrus	L	2.2	−42, 14, −5
	R	2.2	30, 17, −23
Precuneus	R	2.1	3, −64, 31
**PCL:YV Factor 2 intra-network connectivity effects: positive**			
Medial frontal gyrus	L	4.3	−9, −1, 70
	R	2.0	6, 29, 52
Inferior temporal gyrus	L	3.3	−48, −1, −38
Culmen	L	3.2	−6, −46, −2
Inferior frontal gyrus	L	3.1	−33, 14, −17
Caudate	R	2.6	15, 14, 13
	L	2.5	−15, 26, −8
Middle temporal gyrus	L	2.5	−51, −25, −14
Parahippocampal gyrus	L	2.4	−9, −46, 1
Postcentral gyrus	R	2.3	48, −34, 37
Supramarginal gyrus	L	2.2	−42, −49, 37
Medial frontal gyrus	R	2.2	6, 26, 49
Extra-nuclear	L	2.1	−9, −4, −11
Uncus	R	2.1	36, −7, −35
Inferior parietal lobule	R	2.0	48, −43, 46
Middle frontal gyrus	R	2.0	42, 50, 10
**PCL:YV Factor 2 intra-network connectivity effects: negative**			
Caudate	L	2.9	−18, 8, 13
Anterior cingulate	L	2.6	−3, 5, −11
Superior temporal gyrus	L	2.5	−33, 11, −41
Middle temporal gyrus	L	2.5	−48, 2, −20
Supramarginal gyrus	L	2.1	−57, −43, 37
	R	2.5	54, −46, 34
Postcentral gyrus	L	2.0	−63, −28, 37
	R	2.4	60, −19, 31
Temporal pole	L	2.1	−45, 2, −23
Parahippocampal gyrus	L	2.1	−24, −10, −26
Medial frontal gyrus	L	2.0	−9, 62, −2
Inferior parietal lobule	R	2.0	60, −49, 43
**PCL:YV Total intra-network connectivity effects: positive**			
Middle temporal gyrus	L	3.5	−48, 2, −38
Superior frontal gyrus	R	3.2	3, 29, 49
Caudate	R	3.2	21, 20, 7
	R	3.2	18, 17, 7
	L	2.7	−15, 26, −8
Inferior frontal gyrus	L	3.1	−33, 14, −17
Inferior temporal gyrus	L	2.8	−45, −1, −41
Superior temporal gyrus	L	2.7	−51, 11, −20
Middle frontal gyrus	R	2.5	42, 47, −8
Lentiform nucleus	R	2.5	27, −7, −8
Medial frontal gyrus	L	2.1	−9, 56, −2
	R	2.2	6, 26, 49
Supramarginal gyrus	L	2.0	−42, −49, 37
**PCL:YV Total intra-network connectivity effects: negative**			
Superior temporal gyrus	L	3.3	−33, 11, −41
	R	2.1	51, −1, −11
Parahippocampal gyrus	L	3.0	−24, −10, −29
Inferior frontal gyrus	R	2.7	30, 17, −23
Lentiform nucleus	R	2.7	18, 11, −8
Superior frontal gyrus	R	2.6	9, 41, 49
Temporal pole	L	2.5	−30, 8, −41
Caudate	L	2.5	−21, 8, 16
Medial frontal gyrus	L	2.5	−6, 38, 40
Middle frontal gyrus	R	2.3	33, 53, −11
Anterior cingulate	L	2.3	−3, 44, 4
Postcentral gyrus	L	2.2	−60, −28, 37
Precuneus	R	2.1	3, −67, 31

Table shows all significant clusters with a T > 2 that emerge at a p < 0.05 level.

^*^Survive FDR correction at a p < 0.05 level out of networks of interest.

^†^Survive FDR correction at a p < 0.05 level in a whole-brain analysis.

#### 3.4.2. PCL:YV Factor 2 scores

PCL:YV Factor 2 scores were associated with altered intra-network connectivity in regions within all *a priori* networks of interest (see Table 2). Specifically, Factor 2 related increases of intra-network connectivity were found in the medial, middle, and inferior frontal gyrus, middle and inferior temporal gyrus, culmen, caudate, parahippocampal gyrus, postcentral gyrus, supramarginal gyrus, uncus, extra-nuclear regions, and the inferior parietal lobule. Factor 2 related decreases of intra-network connectivity were found in the caudate, anterior cingulate, superior and middle temporal gyrus, supramarginal gyrus, postcentral gyrus, temporal pole, parahippocampal gyrus, medial frontal gyrus, and the inferior parietal lobule (see Table 2). After correction for FDR and controlling for age and IQ, neither *a priori* nor whole brain results emerged for PCL:YV Factor 2 scores.

#### 3.4.3. PCL:YV Total scores

PCL:YV Total scores were associated with altered intra-network connectivity in regions within all *a priori* networks of interest (see Table 2). Specifically, PCL:YV Total score related increases of intra-network connectivity were found in the middle, inferior, and superior temporal gyrus, superior, inferior, middle, and medial frontal gyrus, extra-nuclear and sub-gyral regions, caudate, lentiform nucleus, and supramarginal gyrus. PCL:YV Total score related decreases of intra-network connectivity were found in the superior temporal gyrus, parahippocampal gyrus, inferior, superior, medial, and middle frontal gyrus, lentiform nucleus, temporal pole, anterior cingulate, postcentral gyrus, and precuneus (see Table 2). After correction for FDR and controlling for age and IQ, neither *a priori* nor whole brain results emerged for PCL:YV Total scores.

### 3.5. Inter-network connectivity

**Figure 5:**
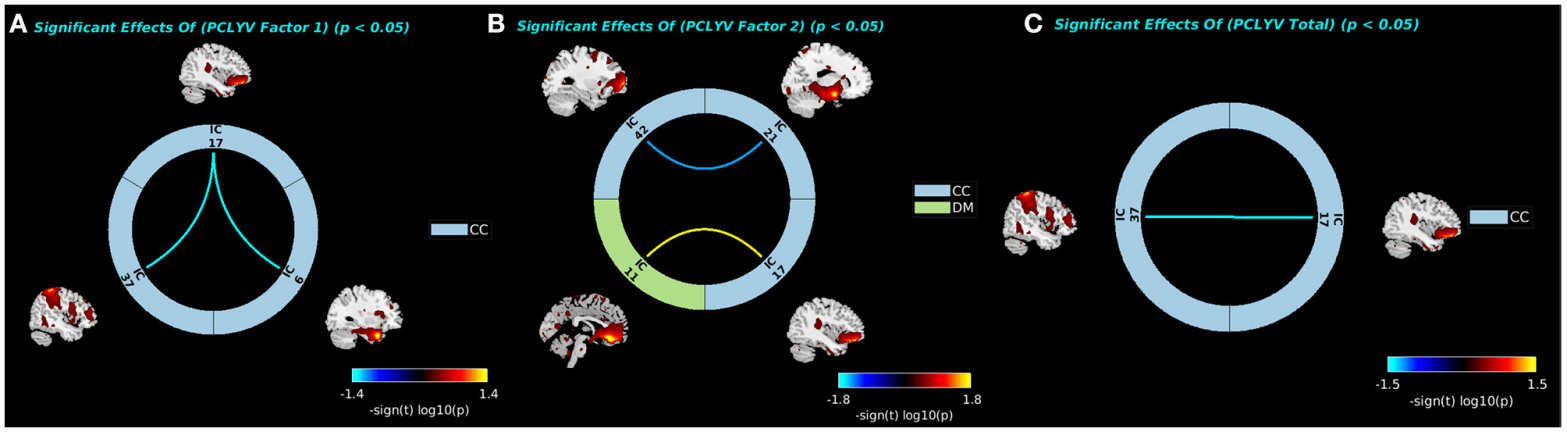
The significant associations between FNC values and PCL:YV Factor 1, Factor 2, and Total scores are shown with connecting curves between networks displayed as –sign(*t*) log_10_(*p*), color bars ranging from −1.4 to 1.4, −1.8 to 1.8, and −1.5 to 1.5, respectively. Red and orange colors correspond to positive correlations; and dark and light blue colors correspond to negative correlations. **(A)** Factor 1 effects showing negative associations between the pars orbitalis (IC17, CC) and both the precuneus (IC37, CC) and temporal pole (IC6, CC). **(B)** Factor 2 effects showing negative associations between the aPFC (IC42, CC) and the amygdalae (IC21, CC), and positive associations between the OFC (IC11, DM) and the pars orbitalis (IC17, CC). **(C)** PCL:YV Total effects showing negative associations between pars orbitalis (IC17, CC) and the precuneus (IC37, CC).

PCL:YV Factor 1 scores were associated with decreased sFNC between the pars orbitalis (IC17, CC) and both the precuneus (IC37, CC) and temporal pole (IC6, CC: see Figure 5A). PCL:YV Factor 2 scores were associated with decreased sFNC between the aPFC (IC42, CC) and the amygdalae (IC21, CC), and increased sFNC between the OFC (IC11, DM) and the pars orbitalis (IC17, CC: see Figure 5B). PCL:YV Total scores were associated with decreased sFNC between the pars orbitalis (IC17, CC) and the precuneus (IC37, CC: see Figure 5C). There were no significant *a priori* nor whole-brain associations between PCL:YV scores and sFNC that survived FDR correction while controlling for age and IQ.

## 4. Discussion

The purpose of this study was to assess abnormalities in resting-state measures related to psychopathic traits in a sample of high-risk adolescent girls. We found that psychopathic traits (assessed via the PCL:YV) were associated with altered functional connectivity and ALFF during a resting-state fMRI experimental paradigm. Consistent with previous research performed in high-risk adolescent boys and adult men and women, PCL:YV scores were associated with altered ALFFs, and inter-/intra-network connectivity across multiple domains, with the majority of effects occurring in limbic, paralimbic, and default mode network regions (i.e., temporal poles, amygdalae, caudate/putamen, orbitofrontal cortex, dorsomedial prefrontal cortex, posterior cingulate cortex, and precunei: Thijssen and Kiehl, 2017; Allen et al., 2022b).

PCL:YV scores were associated with increased ALFFs in low- and high-frequency bands and reduced ALFFs in mid- frequency bands across regions in the CC, DM, SC, SM, and VI domains, with the most robust effects occurring in the PCC, primary motor cortex, and secondary visual cortex. These regions have been commonly implicated in previously published studies relating psychopathic traits to altered resting-state FNC in incarcerated adolescent boys (Cohn et al., 2015; Thijssen and Kiehl, 2017) and community adolescent boy and girl samples (Dugré and Potvin, 2021; Umbach and Tottenham, 2021; Werhahn et al., 2021; Winters et al., 2021). Likewise, the sparse literature investigating resting-state ALFFs and psychopathic traits in incarcerated adolescents have reported largely consistent findings with those obtained in the current study. Specifically, Thijssen and Kiehl (2017) observed that adolescent males scoring high on PCL:YV Factor 1 and Total scores were characterized by increased low-frequency (0–0.01 Hz), decreased low- to mid-frequency (0.05–0.07 Hz), and increased high-frequency (0.20–0.25 Hz) ALFFs in the DM, aligning with the above results: psychopathy-related increased ALFFs at low-frequency bands (0–0.05 Hz) and decreased ALFF at mid/high-frequency bands (0.10–0.15 Hz) in the PCC, and increased ALFFs at high-frequency bands (0.20–0.25 Hz) in the precuneus (see Figures 3A, G: Thijssen and Kiehl, 2017). Importantly, across both studies, these DM effects are accounted by variance in PCL:YV Factor 1, rather than Factor 2. These regions, more generally, are involved in higher-order cognitive processes, such as emotion regulation, movement and action coding/regulation, and attentional modulation (Leech and Sharp, 2014; Kreiman and Serre, 2020; Bhattacharjee et al., 2021).

Accordingly, research suggests that relatively higher-frequency ALFFs, compared to lower-frequency ALFFs, contribute to cognitive processes of higher-order nature (Baria et al., 2011; Craig et al., 2018; though see Biswal et al., 1995). Consistent with similar findings in incarcerated adult women scoring high on psychopathic traits (Allen et al., 2022b), reduced high-frequency ALFFs (e.g., in the PCC) may relate to previously observed deficits characteristic of youth scoring high on psychopathy, such as error-related processing deficits (Maurer et al., 2016). Successful error-related processing depends on the coordination of several brain regions, including psychopathy-related regions as identified above—the primary motor cortex and PCC (Steele et al., 2014). Furthermore, adolescent girls scoring high on psychopathic traits have been previously characterized by altered BOLD reactivity to stimuli featuring facial expressions in the secondary visual cortex, a region also showcasing altered mid- to high-frequency ALFFs related to psychopathic traits within our sample (Fairchild et al., 2014). Finally, and opposite to the deficit hypothesis, some research suggests that increased low-frequency ALFFs may correspond to refined neural efficiency (Biswal et al., 1995). Given findings suggesting that adolescent girls and adult women scoring high on psychopathic traits do not exhibit the same response perseveration deficits as comparable men (Vitale and Newman, 2001; Vitale et al., 2005), the increase in low-frequency ALFFs in regions implicated in response perseveration (i.e., the PCC), may reflect markers of increased neural efficiency (Ersche et al., 2011; Yang et al., 2011). One mechanism that may be giving rise to the observed ALFF effects is altered brain structure. Given that ALFF has been found to vary based on underlying structural differences (Qing and Gong, 2016), the observed effects (e.g., psychopathy related altered ALFF in the OFC) may be related to previously observed structural deficits in the majority of the present sample (Cope et al., 2014).

We also observed that adolescent girls scoring high on psychopathy were characterized by altered inter- and intra-network connectivity in CC and DM networks, with effects in the PFC, precuneus, temporal poles, amygdalae, dmPFC, and left ITG, with the most robust effects occurring in the latter two regions. Specifically, adolescent girls scoring high on psychopathic traits were characterized by increased intra-network functional connectivity within Component 12 (dmPFC) and Component 46 (left ITG), regions consistent with previous analyses in incarcerated adolescents (Chen et al., 2015; Cohn et al., 2015; Thijssen and Kiehl, 2017) and community adolescents (Dugré and Potvin, 2021; Thijssen et al., 2021; Umbach and Tottenham, 2021; Werhahn et al., 2021; Winters et al., 2021). Likewise, consistent with the present analyses and Thijssen and Kiehl (2017), psychopathy-related intra-network connectivity effects that survived FDR correction were constrained to PCL:YV Factor 1, but did not extend to PCL:YV Factor 2 or Total scores. Due to the important role that prefrontal and temporal regions play in emotional regulation, the altered intra-network effects observed in the dmPFC may indicate delayed—or altered—maturation between these circuits and subcortical regions involved in emotional processing (Rubia, 2013; Chen et al., 2015; Morawetz et al., 2016; Dugré and Potvin, 2022), potentially leading to the higher occurrence of antisocial actions associated with psychopathy.

Broadly, our investigation into the relationship between resting-state neurobiological alterations and psychopathic traits in incarcerated adolescent girls underscores two points. First, our results highlight the importance of considering multiple approaches to estimating resting-state alterations on a local (i.e., ALFFs and intra-network connectivity) and global (inter-network connectivity) scale, as these complimentary neurobiological measures are likely to account for alternative types of variance in explaining behavioral traits (see Thijssen and Kiehl, 2017). Second, our results suggest that altered resting-state functional connectivities associated with psychopathic traits present similarly in incarcerated adolescent girls and incarcerated adult women and adolescent boys (Allen et al., 2022b), potentially identifying stable markers for work seeking to predict subsequent antisocial actions utilizing brain-based metrics (Aharoni et al., 2013, 2022; Allen et al., 2022a). More specifically, psychopathy related paralimbic and default mode network alterations in the form of increased low-frequency ALFFs, decreased mid-frequency ALFFs, and increased high-frequency ALFFs were identified across adolescent and adult samples, suggesting that these neurobiological/trait correlates may be stable across development (Thijssen and Kiehl, 2017; Allen et al., 2022b). While the present work adds to a growing literature showcasing consistency in neurobiological alterations from youth to adulthood, future research stands to further explore how these neurobiological correlates may remain or differ from adolescence to adulthood in longitudinal samples of high-risk women.

### 4.1. Study limitations and future research

A number of limitations must be considered alongside the results presented. It is worth noting the small sample size (*n* = 40) compared to other similar analyses (Cohn et al., 2015; Thijssen and Kiehl, 2017; Espinoza et al., 2018; Allen et al., 2022b), potentially casting concerns on the reliability of the results presented. While this shortcoming is likely a side-effect of the low base rate of incarcerated adolescent girls compared to boys in forensic institutions, future studies could consider collaborative efforts that span multiple institutions and samples in order to strengthen the conclusions that can be drawn from the analyses. Additionally, another potential limitation of this study is the length of resting-state scan for the FNC measures being investigated (i.e., static FNC vs. dynamic FNC). While some research suggests that resting-state scans longer than 5-min are necessary to ensure high stability RSNs (Birn et al., 2013), other research finds shorter length scans adequate (Allen et al., 2011a; Espinoza et al., 2018, 2019; Duda et al., 2023). Static FNC, as compared to dynamic FNC, entails a number of assumptions regarding the coherence of the RSN relationships across the 5-min scan, thus, future work should consider exploring variable sliding window dFNC approaches in investigating the stability of resting-state alterations associated with psychopathic traits in incarcerated samples. Likewise, combined analysis of resting-state alterations with task-based scans (such as impulsivity or socioemotional processing tasks) should be considered in future work to not only test the generalizability of neurobiological correlates associated with psychopathic traits, but also corresponding functional and behavioral deficits that may differ during and through development. Thus, more work, and larger samples, are needed to probe the relationships between various functional activity and connectivity measures as they relate to psychopathic traits in incarcerated adolescent women.

### 4.2. Conclusion

Our results suggest that psychopathic traits among incarcerated adolescent girls are associated most robustly with altered intra-network amplitude of low-frequency fluctuations—primarily that of increased low-frequency and decreased mid- to high-frequency fluctuations—and connectivity across multiple networks including paralimbic and default mode network regions, including the PCC. These results, and their relative consistency to similar findings in incarcerated adult women and adolescent boys scoring high on psychopathy (Thijssen and Kiehl, 2017; Allen et al., 2022b), suggest stable neurobiological correlates of psychopathic traits across development. To our knowledge, this is the first study to date on the association of psychopathic traits and intrinsic RSN alterations in incarcerated high-risk adolescent girls.

## Data availability statement

The datasets presented in this article are not readily available because of the potential for personal identification of participants in the present sensitive population (incarcerated adolescent girls). Interested parties should contact the corresponding author, KK for the data used in this report. Requests to access the datasets should be directed at: KK, kkiehl@mrn.org.

## Ethics statement

The studies involving human participants were reviewed and approved by Institutional Review Board of the University of New Mexico. Participants provided written informed consent (if ≥ 18 years or age) or written informed assent and parent/guardian written informed consent (if <18 years of age).

## Author contributions

CA: data curation, methodology, writing–original draft, formal analysis, and writing–review and editing. JM: data curation, methodology, writing-original draft, and writing-review and editing. AG, BE, NA, CH, KH, and KK: data curation, methodology, and writing-review and editing. EA and VC: methodology and writing-review and editing. All authors contributed to the article and approved the submitted version.

## Footnotes

^1^Previous analyses in a subset of the present sample demonstrate demographic similarity to adolescent boys scanned at the same facility in terms of age, IQ, substance dependence, psychopathic traits, and criminal convictions (Ermer et al., 2013; Cope et al., 2014).
