# Supplementary material for: Psychopathic traits and altered resting-state functional connectivity in incarcerated adolescent girls
Source: Front Neuroimaging. 2023 Aug 4;2:1216494. doi: 10.3389/fnimg.2023.1216494 (PMC10406221; doi:10.3389/fnimg.2023.1216494)

Supplementary Material

Psychopathic traits and altered resting-state functional connectivity in incarcerated adolescent girls

Corey H. Allen^1^*, J. Michael Maurer^1^, Aparna R. Gullapalli^1^, Bethany G. Edwards^1^, Eyal Aharoni^2^, Carla L. Harenski^1^, Nathaniel E. Anderson^1^, Keith A. Harenski^1^, Vince D. Calhoun^3,4,5^ & Kent A. Kiehl^1,6*^

*** Correspondence:**Dr. Corey H. Allen & Dr. Kent A. Kiehl
callen@mrn.org (CHA); kkiehl@mrn.org (KAK)

# Supplementary Figures and Tables

## Table S1. Correlation Matrix

|  | 1 | 2 | 3 | 4 | 5 | 6 | 7 | 8 | 9 | 10 |
| --- | --- | --- | --- | --- | --- | --- | --- | --- | --- | --- |
| 1. Age | — |  |  |  |  |  |  |  |  |  |
| 2. IQ | .011 | — |  |  |  |  |  |  |  |  |
| 3. SUD | .077 | 0.332* | — |  |  |  |  |  |  |  |
| 4. PCL:YV Factor 1 | -.265† | .040 | -.036 | — |  |  |  |  |  |  |
| 5. PCL:YV Factor 2 | -.366* | -.215 | .104 | .605*** | — |  |  |  |  |  |
| 6. PCL:YV Total | -.321* | -.100 | .057 | .886*** | .882*** | — |  |  |  |  |
| 7. Anxiety | -.113 | .265 | .044 | -.079 | -.204 | -.156 | — |  |  |  |
| 8. Mood | .112 | .519** | .511** | -.063 | -.026 | -.030 | .327* | — |  |  |
| 9. PTSD | .064 | .027 | -.062 | -.263 | .051 | -.107 | -.240 | -.027 | — |  |
| 10. ADHD | -.211 | .070 | -.074 | -.097 | .134 | -.013 | -.164 | .101 | .364* | — |
| Correlations with SUD, Anxiety, Mood, PTSD, and ADHD reflect a subsample of *n* = 39. † p < 0.05 (one-tailed), *p < 0.05, **p < 0.01, and ***p < 0.001. | | | | | | | | | | |

## Table S2. Resting-state networks (RSNs) domain names, IC numbers, and MNI peak coordinates

| **RSNs and domain names** | **IC Number** | **MNI Peak** |
| --- | --- | --- |
| **Auditory (AU)** |  |  |
| Left superior temporal gyrus | 10 | (-62, -20, 10) |
| **Cerebellar (CB)** |  |  |
| Left posterior lobe | 20 | (-48, -64, -25) |
| Right anterior lobe | 24 | (18, -58, -30) |
| Left posterior lobe | 27 | (-38, -82, -25) |
| **Cognitive Control (CC)** |  |  |
| Left temporal pole | 6 | (-32, 12, -35) |
| Dorsomedial prefrontal cortex | 12 | (0, 44, 55) |
| Left Pars orbitalis | 17 | (-42, 32, -20) |
| Right amygdala | 21 | (18, 0, -20) |
| Left temporal pole | 31 | (-40, 8, -20) |
| Right precuneus | 37 | (48, -44, 60) |
| Left medial temporal gyrus | 41 | (-66, -34, 0) |
| Left anterior prefrontal cortex | 42 | (-24, 58, -5) |
| Left inferior temporal gyrus | 46 | (-54, -30, -20) |
| Supplementary motor area | 50 | (2, 16, 70) |
| Right insula | 51 | (54, 14, -5) |
| Dorsomedial prefrontal cortex | 52 | (0, 26, 45) |
| Dorsomedial prefrontal cortex | 54 | (-18, 32, 60) |
| **Default Mode (DM)** |  |  |
| Right orbitofrontal gyrus | 11 | (0, 36, -15) |
| Right medial temporal gyrus | 34 | (62, -28, -20) |
| Posterior cingulate cortex | 39 | (0, -24, 30) |
| Posterior cingulate cortex | 43 | (0, -52, 15) |
| Precuneus | 45 | (0, -60, 65) |
| Posterior cingulate cortex | 47 | (14, -58, 20) |
| **Subcortical (SC)** |  |  |
| Left putamen | 33 | (-24, 8, 0) |
| **Sensorimotor (SM)** |  |  |
| Left supplementary motor area | 2 | (-56, -10, 30) |
| Right precuneus | 14 | (20, -52, 75) |
| Primary motor cortex | 15 | (2, -26, 75) |
| Right supplementary motor area | 19 | (38, -22, 70) |
| Left primary sensory cortex | 28 | (-48, -36, 60) |
| Left supplementary motor area | 53 | (0, 2, 60) |
| **Visual (VI)** |  |  |
| Right secondary visual cortex | 1 | (24, -94, -20) |
| Right secondary visual cortex | 5 | (36, -94, -5) |
| Right primary visual cortex | 7 | (12, -64, 10) |
| Secondary visual cortex | 13 | (4, -96, 0) |
| Left secondary visual cortex | 18 | (-16, -94, -20) |
| Precuneus | 26 | (0, -82, 45) |
| Left secondary visual cortex | 30 | (-24, -74, -15) |
| Right visual association cortex | 35 | (42, -76, -20) |
| Right angular gyrus | 40 | (60, -60, 20) |

*Note.* RSN network names and domains were determined by peak MNI coordinates and GIFT’s component labeling function.

## Figure S1. Functional network connectivity matrix of the 39 RSNs.


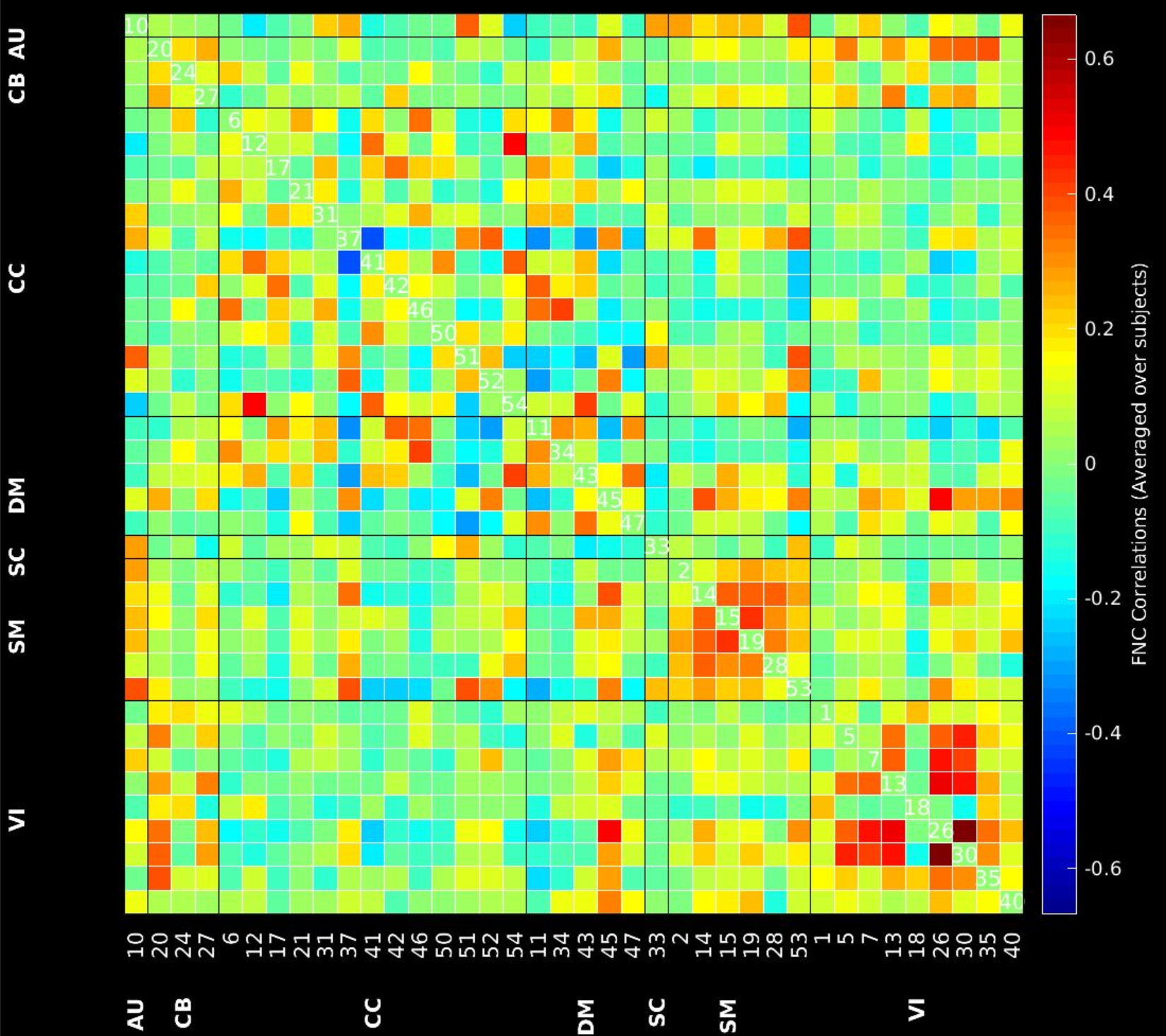

Supplement: Supplementary file 1 [file Data_Sheet_1.docx]
